# Supplementary figures and images for: Early Mediterranean-Based Nutritional Intervention Reduces the Rate of Gestational Diabetes in Overweight and Obese Pregnant Women: A Post-Hoc Analysis of the San Carlos Gestational Prevention Study
Source: Nutrients. 2024 Jul 10;16(14):2206. doi: 10.3390/nu16142206 (PMC11279464; doi:10.3390/nu16142206)

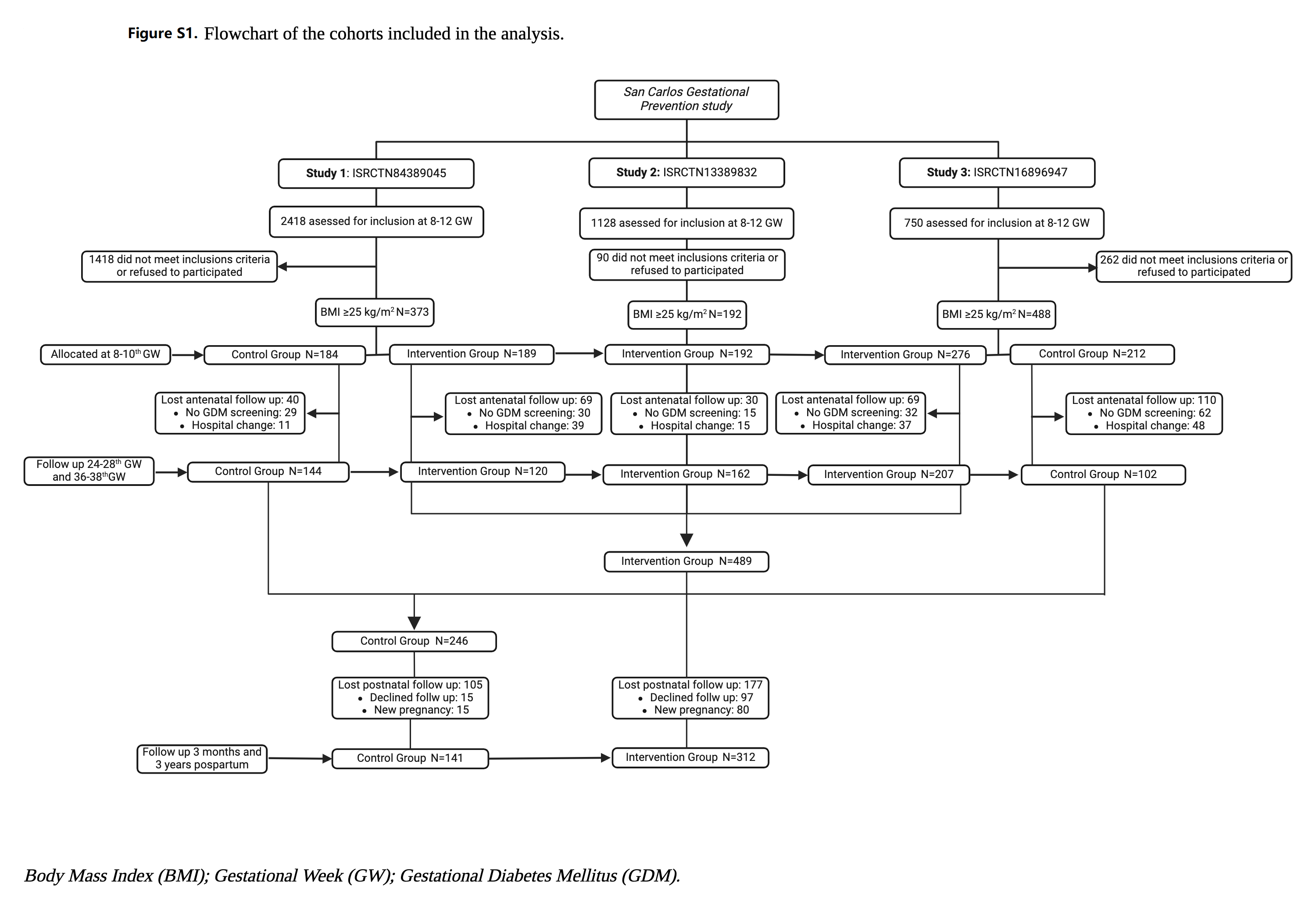

Supplement: Supplementary file 1 [file nutrients-16-02206-s001.zip › nutrients-3052685-supplementary.png]
